# Central Regulation of Metabolism by Protein Tyrosine Phosphatases

**DOI:** 10.3389/fnins.2012.00192

**Published:** 2013-01-07

**Authors:** Ryan C. Tsou, Kendra K. Bence

**Affiliations:** ^1^Department of Animal Biology, School of Veterinary Medicine, University of PennsylvaniaPhiladelphia, PA, USA

**Keywords:** protein tyrosine phosphatase, leptin signaling, insulin signaling, PTP1B, SHP2, PTEN, TCPTP, RPTPe

## Abstract

Protein tyrosine phosphatases (PTPs) are important regulators of intracellular signaling pathways via the dephosphorylation of phosphotyrosyl residues on various receptor and non-receptor substrates. The phosphorylation state of central nervous system (CNS) signaling components underlies the molecular mechanisms of a variety of physiological functions including the control of energy balance and glucose homeostasis. In this review, we summarize the current evidence implicating PTPs as central regulators of metabolism, specifically highlighting their interactions with the neuronal leptin and insulin signaling pathways. We discuss the role of a number of PTPs (PTP1B, SHP2, TCPTP, RPTPe, and PTEN), reviewing the findings from genetic mouse models and *in vitro* studies which highlight these phosphatases as key central regulators of energy homeostasis.

## Introduction

Obesity is a major health issue within the United States with current estimates at greater than one-third of the entire adult US population diagnosed as obese (BMI ≥ 30). When combined with overweight individuals (BMI ≥ 25), the prevalence estimates reach nearly 70% of all US adults (Flegal et al., 2012). It has become increasingly clear that obesity is not only a disorder of peripheral physiology, but also a disease of the central nervous system (CNS). Numerous cellular signals play a role in the central regulation of body weight which can affect feeding and metabolism, and dysregulation of their associated signaling pathways can result in a bias toward weight gain. Phosphorylation is an important regulatory mechanism of intracellular signaling pathways; the opposing actions of kinase and phosphatase activity determine the phosphorylation status of signaling components. Protein tyrosine phosphatases (PTPs) dephosphorylate phosphotyrosyl residues on a variety of receptor and non-receptor substrates. Within the past decade, a number of PTPs [PTP1B, SHP2, TCPTP, Receptor-type protein tyrosine phosphatase epsilon (RPTPe), and PTEN] have been implicated in the central regulation of metabolism. This review will focus on these PTPs in the CNS and explore their roles in regulating metabolism via interactions with the leptin and insulin signaling pathways.

## Leptin and Insulin Signaling: Regulation by Tyrosine Phosphorylation

Two metabolically relevant signaling pathways in the CNS are the leptin and insulin signaling pathways. Both of these pathways are regulated largely by the reciprocal action of protein tyrosine kinases (PTKs) and PTPs. Leptin is an adipocyte-derived hormone which acts in the brain to control energy homeostasis. Within the hypothalamus, leptin inhibits the orexigenic agouti-related protein/neuropeptide Y-expressing (AgRP/NPY) neurons and stimulates the anorexigenic proopiomelanocortin (POMC)-expressing neurons, reducing food intake, and increasing energy expenditure. Leptin signals through its receptor LepRb, a member of the type-1 cytokine receptor family, which is associated with the tyrosine kinase Janus kinase 2 (JAK2). When leptin binds LepRb, JAK2 becomes activated, autophosphorylates, and phosphorylates several tyrosine residues along the intracellular tail of the receptor. These include Y985, Y1077, and Y1138 which, when phosphorylated, allow for recruitment of downstream signaling effectors (reviewed in Bjørbaek and Kahn, 2004; Wauman and Tavernier, 2011). Phosphorylation of these key tyrosine residues along the LepRb activates separate arms of the leptin signaling pathway, each contributing to leptin’s metabolic effects (Table 1). Recently, the role of PTPs as regulators of central leptin signaling has come to light, and a number of PTPs have been shown to interact with LepRb signaling, positively or negatively regulating its downstream signaling capacity.

**Table 1:** **Summary of key tyrosine residues of leptin/insulin signaling components and their cellular/metabolic functions**.

Leptin signaling component/tyrosine involved	Cellular/metabolic function	PTP involved/direction of regulation	Select references
JAK2 Y1007/Y1008	Necessary PTK for phosphorylation of intracellular tail of receptor	PTP1B(−)	Myers et al. (2001), Zabolotny et al. (2002), Cheng et al. (2002)
	Involved in leptin-induced PI3K signaling via IRS activation	RPTPe(−)	Rousso-Noori et al. (2011)
LepRb Y985	Binding site for SHP2. Involved in activation of MAPK pathway	SHP2(+)	Björnholm et al. (2007), Bjorbak et al. (2000)
	Binding site for SOCS3. Involved in negative feedback regulation of LepRb signaling	
LepRb Y1077	Binding site for STAT5. Involved in energy homeostasis/thermal regulation	ND	Mütze et al. (2007), Gong et al. (2007)
LepRb Y1138	Binding site for STAT3. Involved in energy homeostasis/melanocortin production	ND	Bates et al. (2003)
STAT3 Y705	Transcription factor necessary for leptin-induced changes in gene expression	TCPTP(−)	Loh et al. (2011)

**Insulin signaling component/tyrosine involved**	**Cellular/metabolic function**	**PTP involved/direction of regulation**	**Select references**

Insulin receptorβ Y1158	Receptor tyrosine kinase catalytic domain	SHP2(±); RPTPe(−)	Kharitonenkov et al. (1995), Møller et al. (1995)
Insulin receptorβ Y1162/Y1163	Receptor tyrosine kinase catalytic domain	PTP1B(−); TCPTP(−); RPTPe(−)	Salmeen et al. (2000), Galic et al. (2003), Andersen et al. (2001)
IRS 1/2 various Y residues	Necessary effector for protein-complex formation and eliciting downstream PI3K and MAPK signaling	PTP1B(−); SHP2(±)	Goldstein et al. (2000), Asante-Appiah and Kennedy (2003)

*The PTPs involved in regulation at each site are listed and include PTPs that are currently known to have a role in the CNS regulation of energy balance. The direction of PTP regulation indicates whether the specified PTP promotes (+) or inhibits (−) leptin or insulin signaling. ND, not determined*.

Insulin is a pancreas-secreted hormone classically studied for its ability to stimulate uptake of glucose and nutrients into peripheral target tissues. In the CNS however, insulin, like leptin, has been shown to suppress body weight and food intake (Woods et al., 1979; Air et al., 2002) and affect neuropeptide gene expression and hypothalamic neuron activity (Schwartz et al., 1992; Benoit et al., 2002; Choudhury et al., 2005). Similar to leptin signaling, insulin receptor (IR) signaling relies on phosphotyrosyl residues to elicit its downstream effects. In contrast to LepRb however, the IR has intrinsic tyrosine kinase activity; upon ligand binding, the transmembrane β subunits of the IR autophosphorylate on tyrosines Y1158, Y1162, and Y1163, recruiting downstream effector proteins including insulin receptor substrate (IRS). IRS becomes phosphorylated by IR on various Y residues and can activate a number of downstream signaling pathways including the phosphoinositide-3 kinase (PI3K)-Akt and MAPK pathways (reviewed in White, 1997). Historically, much of what is known regarding the cellular mechanisms underlying insulin signaling has been studied within the periphery; the connection between CNS insulin signaling and energy homeostasis is less well defined. Neuron-specific IR knockout mice demonstrated genetic evidence that central IR signaling plays an important role in regulating body weight (Brüning et al., 2000). Furthermore, pharmacological studies have revealed that the reduction of food intake by acute central insulin administration is PI3K-dependent (Niswender et al., 2003), and *in vitro* models have identified the MAPK pathway as important for insulin’s ability to regulate NPY/AgRP gene expression (Mayer and Belsham, 2009). In contrast to the relatively recent emergence of PTPs as regulators of central leptin signaling, many PTPs have been historically linked to the insulin signaling pathway, including PTP1B, SHP2, TCPTP, and PTEN. In spite of this, their roles as regulators of central insulin signaling have yet to be studied in depth.

Separate from the classical JAK2-STAT3, PI3K-Akt, and MAPK pathways, leptin and insulin may mediate intracellular signaling via other signaling proteins. For example, Tub is a protein primarily expressed in the CNS which has been shown to be phosphorylated by IR and JAK2 *in vitro* (Kapeller et al., 1999). Targeted deletion of the *tub* gene in mice results in mature-onset obesity with noticeable weight gain beginning at 8 weeks of age (Stubdal et al., 2000). More recently, Tub has been implicated in central insulin and leptin signaling *in vivo*; Tub appears to act downstream of hypothalamic leptin and insulin signaling, potentially as a transcription factor. Moreover, PTP1B may be a negative regulator of CNS leptin and insulin signaling via direct dephosphorylation of Tub (Prada et al., 2012). Thus, central leptin and insulin can affect novel downstream effectors in addition to JAK2/STAT3-, PI3K-, and MAPK-dependent signaling pathways, and these distinct signaling proteins may be regulated by PTPs such as PTP1B.

## The PTP Superfamily and the Prototypic PTP1B

The PTP superfamily consists of approximately 100 different PTPs which can be divided into two groups: classical phospho-tyrosine specific phosphatases and dual-specificity phosphatases. Classical PTPs only dephosphorylate phospho-tyrosine residues while dual-specificity phosphatases can also dephosphorylate serine and threonine residues. The classical, phospho-tyrosine specific phosphatases can further be subdivided into receptor-like and soluble, non-transmembrane PTPs depending upon cellular localization. All PTPs contain the (I/V)HCXXGXXR(S/T) active-site motif with a conserved cysteine residue which is essential for phosphate recognition and catalysis (reviewed in Tonks, 2003, 2006). Structural diversity within domains attached to the core recognition motif defines a PTP’s substrate specificity and cellular localization. Much of the understanding of PTP structure and function has come from studies of protein tyrosine phosphatase 1B (PTP1B), the first PTP to be purified and to have its crystal structure determined (Tonks et al., 1988; Barford et al., 1994). Through the use of site-directed mutagenesis, catalytically inactive “substrate-trapping” mutants were generated in order to identify PTP1B targets (Flint et al., 1997). Substrate-trapping findings combined with structural evidence led to the determination that PTP1B preferentially binds to the tandem phospho-tyrosine motif (E/D)-pY-pY-(R/K), due to the presence of an additional phospho-tyrosine recognition site adjacent to its catalytic site. PTP1B was shown to bind and dephosphorylate JAK2 preferentially at pY1007/pY1008 (Myers et al., 2001), and the IR at pY1162/pY1163 (Salmeen et al., 2000). Furthermore, it is also likely that PTP1B can dephosphorylate IRS 1 (Goldstein et al., 2000).

PTP1B was first implicated in the control of energy homeostasis through the generation of PTP1B−/− mice (Table 2). PTP1B−/− mice display resistance to diet-induced obesity and reduced adiposity due to increased energy expenditure, and improved peripheral glucose homeostasis as demonstrated by glucose and insulin tolerance tests (Elchebly et al., 1999; Klaman et al., 2000). On a high-fat diet, PTP1B−/− mice have decreased serum leptin levels, suggesting that they might be leptin hypersensitive. Indeed, PTP1B-deficiency improves leptin sensitivity both *in vitro* and *in vivo* (Cheng et al., 2002; Zabolotny et al., 2002). Interestingly, compound deletion of both leptin and PTP1B (*ob/ob*: *Ptpn1*−/−) results in mice with attenuated weight gain in comparison to leptin-deficient *ob/ob* mice, suggesting PTP1B may regulate leptin-independent (e.g., central insulin signaling) pathways important in the regulation of body weight (Cheng et al., 2002).

**Table 2:** **Summary of CNS PTP-genetic mouse models and their associated metabolic phenotypes**.

PTP mouse model	Body weight/adiposity phenotype	Leptin sensitivity	Glucose homeostasis	Reference
Global PTP1B−/− (whole body)	Decreased	Increased	Improved GTT Improved ITT	Klaman et al. (2000), Elchebly et al. (1999)
Neuron-specific PTP1B −/− (Nestin-Cre)	Decreased	Increased	Improved GTT Improved ITT	Bence et al. (2006)
POMC neuron-specific PTP1B−/− (POMC-Cre)	Decreased	Increased	Improved GTT Improved ITT	Banno et al. (2010)
LepRb-specific PTP1B−/− (LepRb-Cre)	Decreased	Increased	Improved GTT	Tsou et al. (2012)
Forebrain-specific SHP2−/− (CaMKIIα-Cre)	Increased	Decreased	(Fed) Hyperglycemia Hyperinsulinemia Fatty liver	Zhang et al. (2004)
Brain-specific SHP2−/− (CRE3)	Increased	Decreased	Impaired glucose tolerance Hyperinsulinemia	Krajewska et al. (2008)
POMC neuron-specific SHP2−/− (POMC-Cre)	Increased	Decreased	Hyperinsulinemia Impaired glucose tolerance	Banno et al. (2010)
Forebrain-specific dominant-active SHP2 (D61A)	Decreased in females	Increased in females	Improved GTT females Improved ITT females	He et al. (2012)
Neuron-specific TCPTP−/− (Nestin-Cre)	Decreased	Increased	Improved GTT Improved ITT	Loh et al. (2011)
Neuron-specific TCPTP−/−:PTP1B−/− (Nestin-Cre)	Decreased (additive effects)	Increased (additive effects)	Improved ITT (additive effects)	Loh et al. (2011)
Global RPTPe−/− (whole body)	Decreased in females	Increased in females	Improved GTT Improved ITT	Rousso-Noori et al. (2011)
LepRb-specific PTEN−/− (LepRb-Cre)	Decreased	Increased leptin-induced PI3K pathway	Improved GTT Improved ITT Decreased serum insulin	Plum et al. (2007)
LepRb-specific PTEN overexpression (LepRb-Cre)	No change	Decreased leptin-induced PI3K pathway	Fatty liver	Warne et al. (2011)
POMC-PTEN−/− (POMC-Cre)	Increased, sex and diet-dependent	Decreased food intake suppression	ND	Plum et al. (2006)
VMH-specific PTEN−/− (SF1-Cre)	Increased	ND	ND	Klöckener et al. (2011)

*ND, not determined*.

Generation of a number of tissue-specific PTP1B-deficient mouse models has provided insight into PTP1B’s central metabolic effects, separate from its functions at the periphery. Overall these studies suggest that the reduction in body weight and adiposity afforded by PTP1B-deficiency are a result of central loss of PTP1B. Brain-specific PTP1B−/− mice (generated with a nestin-cre transgenic line) display improved body weight and adiposity compared to controls on both chow and HFD; these mice have decreased food intake, increased energy expenditure, and are leptin and insulin hypersensitive (Bence et al., 2006). Mice deficient in PTP1B specifically within POMC neurons (POMC-PTP1B−/−) also show decreased body weight and adiposity, albeit only when fed a high-fat diet, due to increased energy expenditure. Notably, on a chow diet POMC-PTP1B−/− mice show improved glucose tolerance and insulin sensitivity even when controlled for body weight and adiposity, indicating that central PTP1B can regulate peripheral glucose homeostasis (Banno et al., 2010). POMC-PTP1B−/− mice also display enhanced leptin sensitivity within the hindbrain and a heightened homeostatic response to cold exposure (De Jonghe et al., 2011, 2012). More recently, LepRb-PTP1B−/− mice were generated using a LepRb-cre line in which Cre recombinase is specifically expressed within all LepRb-expressing neurons; mice with LepRb-specific PTP1B-deficiency show reduced body weight and adiposity compared to wild type controls on both chow and HFD. These mice have slightly decreased food intake and slightly elevated core temperature compared to controls. Consistent with past findings, LepRb-PTP1B−/− mice are leptin hypersensitive and show improved glucose homeostasis (Tsou et al., 2012). When compared to a parallel line of global PTP1B−/− mice, LepRb-PTP1B−/− mice on a high-fat diet show similar reductions in body weight and leptin sensitivity, suggesting that the majority, if not all, of these metabolic effects are due to a lack of PTP1B within this subset of neurons. Whether any additional pathways aside from those involving LepRb/JAK2 are involved in mediating PTP1B’s effects *in vivo* remains to be determined.

In contrast to mouse models of central PTP1B-deficiency, liver- and muscle-specific PTP1B deletion results in enhanced peripheral insulin sensitivity without effects on body weight or adiposity (Delibegovic et al., 2007, 2009). Adipocyte-specific PTP1B deletion has no effect on body weight or total adiposity, but increases adipocyte size, circulating glucose and leptin levels (Owen et al., 2012). In summary, these findings indicate PTP1B has CNS-specific regulatory effects on body weight mediated via its interaction with leptin signaling, and potentially by other as yet unidentified pathways. In peripheral tissues such as muscle and liver, PTP1B acts as a negative regulator of insulin signaling, resulting in effects on glucose homeostasis. Whether or not PTP1B can regulate CNS insulin signaling to alter energy balance remains unclear and warrants further investigation.

## SHP2: Positive Regulator of Leptin Receptor → MAPK Signaling

SHP2 is an ubiquitously expressed Src homology 2 domain-containing non-transmembrane protein tyrosine phosphatase (Freeman et al., 1992). SHP2 contains two tandem src homology 2 (SH2) domains at its N-terminus which provide support for binding phosphotyrosyl residues. SHP2 has been shown to play a role in numerous growth factor and cytokine-induced signaling pathways and as mentioned earlier within the context of leptin signaling, is a key intermediate for eliciting downstream MAPK signaling (via binding Y985 of LepRb; Banks et al., 2000). Despite its role as a tyrosine phosphatase and early *in vitro* evidence suggesting SHP2 can inhibit JAK2 activation or downregulate STAT3-mediated gene transcription (Carpenter et al., 1998; Li et al., 1999), SHP2 has been shown to ultimately promote leptin signaling within the CNS. Interestingly in spite of its perceived “adaptor” role within leptin signaling, SHP2’s phosphorylation and functional phosphatase activity are required for leptin-induced ERK activation (Bjørbaek et al., 2001). While global SHP2−/− mutation is embryonic lethal in mice (Saxton et al., 1997), in 2004 a forebrain/neuronal-specific SHP2−/− mouse model was generated using the CaMKIIα-cre line, resulting in a pronounced obese phenotype. CaMKIIα-SHP2−/− mice show increased body weight and adiposity compared to wild type controls due to decreased energy expenditure. These mice also display increased serum leptin, disrupted *Npy* mRNA expression in response to fasting, elevated fed blood glucose, and evidence of fatty liver. Additionally, CaMKIIα-SHP2−/− mice show significantly reduced leptin-induced phosphorylated-ERK within the arcuate nucleus, supporting SHP2’s role as a positive regulator of leptin signaling via the MAPK pathway (Zhang et al., 2004). Neuronal SHP2−/− mice developed using a pan-neuronal Cre line, CRE3 (Banares et al., 2005) similarly show an obese phenotype with increased body weight, adiposity, hyperleptinemia, and diabetic symptoms (Krajewska et al., 2008). Interestingly, CRE3-SHP2−/− mice are hyperphagic and show disruptions in CNS leptin signaling distinct from those seen in CaMKIIα-SHP2−/− mice. While CaMKIIα-SHP2−/− mice have reduced leptin-induced ERK activation compared to wild type controls, CRE3-SHP2−/− display elevated basal ERK phosphorylation which is decreased after leptin injection (Krajewska et al., 2008). The phenotypic differences between the CaMKIIα-SHP2−/− and the CRE3-SHP2−/− mouse models may be a result of greater neuronal SHP2 deletion in the pan-neuronal CRE3 line. Using the transgenic CRE3 line, additional leptin-sensitive regions involved in energy balance including the hindbrain are targeted which may affect the resultant phenotype. Furthermore, temporal differences in Cre expression due to different promoters may also have an effect. The CaMKIIα promoter is known to “turn on” Cre expression at P5 whereas the CRE3 line shows Cre expression as early as E11.5. As discussed further below, SHP2 is involved in CNS development, thus temporal differences in SHP2 deletion could affect the development of feeding/satiety-related neuronal circuits. Alternatively, differences in genetic background of the mice in the two studies may account for any phenotypic inconsistencies. Deletion of SHP2 from a specific neuronal population, POMC-expressing cells, results in mice with increased body weight, adiposity, and serum leptin levels on both chow and HFD. Consistent with elevated serum leptin, POMC-SHP2−/− mice display leptin resistance and impaired *Pomc* mRNA expression (Banno et al., 2010). Like the CaMKIIα-SHP2−/− mice, POMC-SHP2−/− mice show no changes in food intake but decreased energy expenditure, likely explaining the increased weight gain. POMC-SHP2−/− mice also show impairments in peripheral glucose homeostasis compared to controls however when matched for body weight and composition, no differences were observed, indicating impairments to glucose homeostasis are secondary to elevated body weight and adiposity of POMC-SHP2−/− mice (Banno et al., 2010).

Recently, a transgenic SHP2 dominant-active (D61A) mutant driven by the CaMKIIα promoter was generated. Given deletion of SHP2 in post-mitotic forebrain neurons confers an obese phenotype, one might expect a gain-of-function mutation to provide an improved metabolic phenotype. Interestingly, only female SHP2^D61A^ mice show resistance toward high-fat diet-induced obesity, while male SHP2^D61A^ mice gain weight similar to wild type controls (He et al., 2012). Female SHP2^D61A^ have decreased food intake, increased energy expenditure, and improved leptin sensitivity compared to controls. Additionally, SHP2 was found to physically associate with estrogen receptor α, and leptin and estrogen stimulation synergistically enhanced ERK phosphorylation in the brains of SHP2^D61A^ mice compared to controls, explaining the observed sex differences (He et al., 2012). The lack of a strong metabolic phenotype in the SHP2-active mutant model suggests SHP2’s ability to positively regulate leptin signaling may reach a “ceiling effect” in its physiological benefit. It appears that enhancing the SHP2 arm of the leptin pathway results in an improved metabolic phenotype in female mice due to coupled action with estrogen, but the effects of SHP2 activation on energy balance independent of estrogen may play a less prominent role than other downstream leptin pathways (e.g., JAK2-STAT3 signaling). Mutation of SHP2’s putative binding site on the LepRb, Y985, results in a lean phenotype in young animals (Björnholm et al., 2007; You et al., 2010), presumably due to the inability of SOCS3 to inhibit leptin signaling. Thus, the obese phenotypes of neuronal SHP2−/− models are possibly due to enhanced SOCS3 inhibition of leptin signaling (via binding Y985) rather than the loss of downstream SHP2-mediated leptin signaling.

In addition to playing a positive role in transmitting downstream LepRb signals, SHP2 has also been implicated in neuronal development via regulation of growth factor pathways. Deletion of SHP2 in neuronal progenitors results in mice with early post-natal lethality and disrupted corticogenesis (Ke et al., 2007), and expression of a dominant negative SHP2 mutant in cultured sympathetic neurons results in decreased neurite outgrowth and survival (Chen et al., 2002). Furthermore, POMC-SHP2−/− mice displayed a reduction in α-melanocyte-stimulating hormone (POMC cleavage product) content in the periventricular nucleus and dorsal medial hypothalamus, indicating SHP2 may influence the development of hypothalamic feeding/satiety projections and circuits (Banno et al., 2010). Overall, these findings suggest that impaired central leptin signaling at least partially underlies the obese phenotypes of CNS-specific SHP2 knockout models. However, given SHP2’s importance in neuronal development, subtle differences in CNS-architecture in regions controlling energy balance may also influence weight gain. In addition, SHP2 has been shown to act as both a positive and negative regulator of insulin signaling in different contexts (Asante-Appiah and Kennedy, 2003); whether SHP2 mediates central effects of insulin on energy balance has not been investigated.

## TCPTP: Negative Regulator of STAT3

T-cell PTP (TCPTP) is a ubiquitously expressed PTP which shares a high-degree of sequence and structure homology with PTP1B. Despite similarities, TCPTP and PTP1B have distinct substrate specificity; TCPTP has been shown to target both JAK1 and JAK3 (Simoncic et al., 2002), whereas PTP1B, as mentioned earlier, directly dephosphorylates JAK2 (Myers et al., 2001; Cheng et al., 2002; Zabolotny et al., 2002). Like PTP1B, TCPTP has also been shown to negatively regulate insulin signaling via dephosphorylation of IRβ at Y1162/Y1163 (Galic et al., 2003), though differential effects on intensity and duration of IR phosphorylation suggest TCPTP and PTP1B have non-redundant influences on insulin signaling (Galic et al., 2005). TCPTP mRNA can be alternatively spliced resulting in two different TCPTP isoforms of different sizes: 48 kDa TC48 and 45 kDa TC45. The TC45 isoform is localized to the nucleus and directly dephosphorylates IL-6-induced pSTAT3 (Yamamoto et al., 2002; Tiganis and Bennett, 2007). Recently, in a comprehensive study by Loh et al. (2011) neuronal TCPTP−/− mice were generated and presented a profound metabolic phenotype, providing *in vivo* evidence that TCPTP can regulate leptin-induced JAK/STAT signaling (Loh et al., 2011). Neuronal TCPTP−/− mice show resistance to diet-induced obesity, decreased adiposity on HFD, and increased energy expenditure. Interestingly, neuronal TCPTP−/− mice on chow have decreased body weight but increased relative adiposity. In spite of increased relative adiposity, neuronal TCPTP−/− mice have decreased circulating leptin levels and improved leptin sensitivity, demonstrated both biochemically (increased leptin-induced pSTAT3 levels in hypothalamus) as well as physiologically (greater suppression of body weight/food intake). Alternatively, pharmacological inhibition of central TCPTP also improves leptin sensitivity in wild type animals. Expression of TC45 substrate-trapping mutant within LepRb-expressing CHO cells results in pSTAT3 accumulation in the nucleus, supporting the role of TCPTP as a regulator of leptin signaling via dephosphorylation of STAT3. Neuronal TCPTP−/− mice also show enhanced peripheral glucose homeostasis as measured by glucose and insulin tolerance tests (Loh et al., 2011).

Like PTP1B, TCPTP is induced by high-fat feeding implicating it in the pathogenesis of leptin resistance and obesity. In mice fed HFD, PTP1B and SOCS3 were induced at 6 weeks of high-fat feeding while TCPTP was induced at 9 weeks. Thus, increased TCPTP may contribute to leptin resistance initially brought on by high levels of PTP1B and SOCS3 (Loh et al., 2011). Since PTP1B and TCPTP act as negative regulators at different points within the leptin signaling pathway, Loh et al. further examined whether both enzymes could additively contribute to leptin resistance. Loh et al. generated neuronal PTP1B−/−:TCPTP−/− double knockouts and found PTP1B−/−:TCPTP−/− mice are leaner on HFD and more leptin hypersensitive than single PTP1B−/− mice, suggesting the combined PTP1B/TCPTP deficiency synergistically sensitizes leptin signaling. These results indicate that both PTP1B and TCPTP can inhibit central leptin signaling via the dephosphorylation of JAK2 and STAT3, respectively and also leave open the possibility that the two PTPs act within the same neuron populations, inhibiting separate components of leptin signaling.

## RPTPe: Negative Regulator of JAK2

Receptor-type protein tyrosine phosphatase epsilon is transmembrane receptor-like PTP. Modification at the transcriptional or post-transcriptional level can produce cytosolic, soluble forms of the enzyme, affecting subcellular localization, and in turn substrate specificity (Andersen et al., 2001; Gil-Henn et al., 2001). Both RPTPe and cytosolic PTPe have been shown to negatively regulate insulin signaling (Møller et al., 1995; Andersen et al., 2001; Aga-Mizrachi et al., 2008), and cytosolic PTPe has been implicated in inhibition of MAPK and JAK/STAT signaling (Tanuma et al., 2000; Toledano-Katchalski et al., 2003). Recently, Rousso-Noori et al. generated RPTPe-deficient mice by targeting the *Ptpre* gene and removing three exons encoding amino acids common to both RPTPe and soluble PTPe. Thus, RPTPe−/− mice are deficient in all forms of the enzyme (Peretz et al., 2000; Rousso-Noori et al., 2011). Although male RPTPe−/− mice display no body weight differences compared to wild type controls, female RPTPe−/− mice show resistance to diet-induced obesity with decreased body weight and decreased adiposity and adipocyte size. In addition to protection from HFD-induced weight gain, female RPTPe−/− mice are resistant to weight gain after ovariectomy. RPTPe−/− mice show no differences in food intake compared to wild type controls, suggesting increased energy expenditure explains the lean phenotype. Indeed, energy expenditure is increased in RPTPe−/− mice compared to controls as determined by increased heat production, oxygen consumption, and carbon dioxide production. RPTPe−/− mice also show enhanced peripheral glucose homeostasis and cellular/physiological leptin sensitivity. Although all RPTPe−/− mice show evidence of improved glucose metabolism, only females show improved leptin sensitivity, consistent with the sex differences in body weight and adiposity. Rousso-Noori et al. examined potential RPTPe substrates within leptin signaling *in vitro* and determined RPTPe directly interacts with JAK2, and that the RPTPe-JAK2 complex coimmunoprecipitates with LepRb. Furthermore, leptin-induced JAK2 activation results in phosphorylation of RPTPe on Y695, enhancing its ability to dephosphorylate JAK2 when compared to the non-phosphorylatable mutant RPTPe Y695F, providing evidence that central leptin signaling can regulate RPTPe activity via JAK2 phosphorylation. Based upon these findings, Rousso-Noori et al. proposed a model in which RPTPe plays a role as a negative feedback regulator of leptin signaling via dephosphorylation of JAK2 (Rousso-Noori et al., 2011).

Despite clear evidence of RPTPe as a JAK2-targeting phosphatase, much remains unknown regarding its ability to centrally regulate energy balance. The improved metabolic phenotype of RPTPe-deficiency shows clear sex differences with females displaying greater metabolic benefits than males. As mentioned earlier at the level of leptin-induced SHP2-ERK activation, estrogen and leptin signaling exhibit substantial cross-talk; in addition to SHP2, STAT3 has been shown to be a target of estrogen receptor signaling (Gao and Horvath, 2008). There is evidence of estrogen enhancing leptin sensitivity (Clegg et al., 2006), therefore one could speculate that the enhanced leptin-induced JAK2-STAT3 signaling in RPTPe-deficient mice may elicit a greater physiological effect within females. Interestingly, mice with PTP1B-deficiency demonstrate the reverse sex effect with males displaying a stronger protective metabolic phenotype despite both PTPs targeting JAK2. It will be interesting to determine the precise pattern of PTP expression within subsets of energy balance neurons; perhaps RPTPe is enriched within estrogen receptor-expressing hypothalamic neurons whereas PTP1B shows a separate pattern of hypothalamic expression. Furthermore, RPTPe has been implicated in neuronal excitability via modulation of voltage-gated potassium channels (Ebner-Bennatan et al., 2012); however, how altered hypothalamic neuron excitability can affect whole body energy homeostasis is an area of continued investigation.

## PTEN: Negative Regulator of PI3K-Akt Signaling

Phosphatase and tensin homolog deleted on chromosome 10, or PTEN, is a dual-specificity protein tyrosine phosphatase best known for its role as a tumor suppressor (Li et al., 1997; Steck et al., 1997). Although PTEN is a member of the PTPase family, its primary physiological target appears to be the phospholipid PIP_3_ rather than a tyrosine-phosphorylated protein (Maehama and Dixon, 1998). Thus, PTEN opposes the action of PI3K by directly dephosphorylating PIP_3_ into PIP_2_, acting as a downstream negative regulator of both leptin and insulin signaling pathways. Despite a clearly defined intracellular role for PTEN opposing leptin- or insulin-stimulated PI3K signaling, its ability to centrally regulate metabolism is less transparent due to contrasting phenotypes of transgenic and mutant mouse models. LepRb-specific deletion of PTEN results in decreased body weight, adiposity, and adipocyte size in mice; food intake is unchanged, and oxygen consumption is increased compared to controls (Plum et al., 2007). Increased leptin-stimulated sympathetic nerve activation and “browning” of perigonadal white adipose tissue in LepRb-PTEN−/− mice also point toward increased energy expenditure as the cause of leanness. The metabolic improvements seen in LepRb-PTEN−/− mice are dependent on the presence of circulating leptin as compound *ob/ob*:LepRb-PTEN−/− mice show a comparable phenotype to *ob/ob* single mutants (Plum et al., 2007). Surprisingly, LepRb-specific overexpression (OE) of PTEN in mice does not result in an obese phenotype; instead, LepRb-PTEN-OE mice display no difference in body weight, adiposity, or leptin levels compared to controls. However, consistent with the findings of Plum et al. (2007), attenuation of leptin-activated PI3K signaling in LepRb-PTEN-OE mice increases liver triglycerides due to a reduction in leptin-stimulated sympathetic nerve activity (Warne et al., 2011). Contrastingly, whole body PTEN-OE in mice results in decreased body weight and adiposity due to increased energy expenditure; however, the enhanced energy expenditure appears to be a cell autonomous effect within hyperactive brown adipocytes rather than CNS-related (Ortega-Molina et al., 2012).

In contrast to LepRb-PTEN−/− mice, POMC neuron-specific PTEN deletion in mice unexpectedly results in a sexually dimorphic, diet-sensitive *increase* in body weight and hyperphagia. Male POMC-PTEN−/− mice show a consistent increase in body weight/adiposity on normal chow but no difference on HFD compared to controls, while female mice only show a significant obese phenotype on HFD. The phenotype of POMC-PTEN−/− mice is a result of decreased POMC neuron excitability due to heightened conductance of PIP_3_-sensitive, hyperpolarizing K_ATP_ channels (Plum et al., 2006). The authors confirm that insulin in fact hyperpolarizes POMC neuron membrane potential and present a model where sensitization of insulin-activated PI3K signaling in POMC neurons via targeted PTEN deletion results in decreased POMC neuron excitability, ultimately promoting weight gain. Interestingly, reduction of POMC neuron PI3K signaling via deletion of the p110α catalytic subunit of PI3K within POMC neurons also increases body weight and adiposity and reduces energy expenditure in mice (Hill et al., 2009). This same discordant trend is observed in targeted deletion of PTEN/p110 in the ventral medial hypothalamus (VMH). PTEN-deficiency in SF1 neurons within the VMH results in increased body weight due to hyperphagia (Klöckener et al., 2011). Klöckener et al. further demonstrate that insulin-responsive SF1 neurons are inhibited by insulin treatment, which is dependent upon K_ATP_ channel conductance. Consistent with those findings, VMH-specific deletion of IR yields a protective metabolic phenotype, and Klöckener et al. provide convincing electrophysiological evidence that POMC neuron activity is enhanced by attenuating insulin-stimulated PI3K signaling in SF1 neurons, suggesting synaptic connectivity between VMH neurons and arcuate POMC neurons. Like the effects seen with POMC neuron-specific p110α deletion, VMH-specific p110α deletion results in increased body weight gain and adiposity on HFD due to decreased energy expenditure; PI3K disruption in VMH also blunted the food intake suppressing effects of leptin (Xu et al., 2010). These studies taken together suggest that the activity of the PI3K pathway, as regulated by PTEN, has multiple and disparate effects on energy homeostasis due to different cellular consequences (e.g., affecting gene transcription or neuronal activity via K_ATP_ channels) and is dependent upon neuronal identity (i.e., leptin- versus insulin-responsive populations). The apparent contradiction of increased PI3K pathway activation (via targeted PTEN deletion) and decreased PI3K activation (via targeted p110α deletion) yielding similar weight gain phenotypes implies that the active PI3K pathway leads to at least two separate outputs which have opposing effects on energy balance. How these distinct outputs ultimately result in the observed metabolic phenotype is unclear, although variations in neuronal cell type could play a role. Recently, Williams et al. demonstrated that leptin- and insulin-responsive POMC neurons are segregated in distinct subpopulations (Williams et al., 2010) which may explain inconsistencies between LepRb- and POMC-specific PTEN knockout models. Furthermore, limitations of current cre/loxp technology leading to unexpected off target deletion (Padilla et al., 2010, 2012) may contribute to the inconsistency between the PTEN mutant mouse phenotypes. Careful dissection of downstream PI3K pathways will be necessary in order to further reveal PTEN’s central metabolic role.

## Perspectives and Conclusion

**Figure 1:**
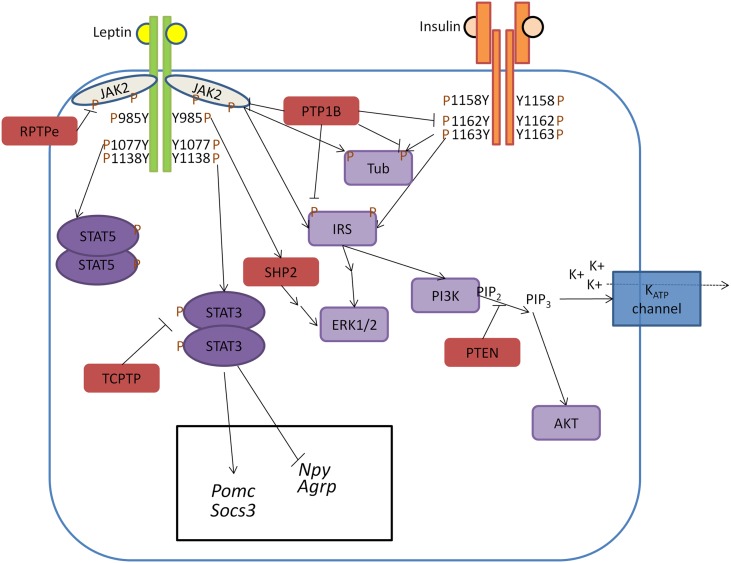
**Model of PTP regulation of central leptin and insulin signaling**. When circulating leptin binds to its receptor LepRb, the associated tyrosine kinase JAK2 autophosphorylates and phosphorylates specific tyrosine residues along the intracellular tail of the LepRb. Phosphorylation of Y985 allows for recruitment of the PTP SHP2 which mediates downstream ERK1/2 signaling, while phosphorylation of Y1138 allows for activation of STAT3 which regulates transcription of key neuropeptides involved in energy homeostasis. Unlike leptin signaling, insulin binding to its receptor results in receptor autophosphorylation at tyrosine residues 1158, 1162, and 1163. This allows for recruitment of the effector IRS, which upon phosphorylation can recruit adaptor molecules and mediate downstream PI3K and ERK1/2 signaling. In contrast to SHP2 which positively regulates leptin signaling, several PTPs can negatively regulate central leptin and insulin signaling. PTP1B inhibits leptin and insulin signaling by dephosphorylating JAK2 and the IR, respectively. Additionally, PTP1B has been implicated in dephosphorylating the downstream leptin/insulin signaling protein Tub. Like PTP1B, RPTPe has been shown to inhibit leptin signaling at the level of JAK2, while TCPTP negatively regulates leptin signaling via dephosphorylation of STAT3. PTEN antagonizes neuronal insulin-induced PI3K signaling via dephosphorylation of the phospholipid PIP_3_ into PIP_2_, resulting in decreased K_ATP_ channel conductance.

Protein tyrosine phosphatases play a major role in regulating pathways downstream of CNS leptin and insulin signaling; however, much is still unknown regarding their ability to centrally regulate energy balance, and a number of open questions remain in the field. Specifically, in regards to insulin signaling, how PTPs interact with IR signaling within the CNS remains largely unknown. Although PTP1B, SHP2, TCPTP, and RPTPe have been shown to negatively regulate IR signaling in the periphery, these PTPs have not been specifically studied within the context of CNS insulin signaling. One can speculate that inhibition of central PTPs may sensitize central insulin signaling and promote weight loss; however, the findings from the PTEN mouse models suggest a more complex role for CNS insulin signaling. In addition to leptin-like food intake reducing effects, central insulin can inhibit neuronal electrical activity which may ultimately promote weight gain. Additional analysis of the roles of specific PTPs in the context of CNS leptin and insulin signaling is required to determine their respective metabolic contribution. Furthermore, what governs PTP specificity when many PTPs seem to regulate the same molecules downstream of leptin/insulin signaling? PTP1B, RPTPe, and TCPTP all target the JAK2/STAT3 pathway, and JAK2 is a direct substrate for both PTP1B and RPTPe (Figure 1). Plasma membrane localization and tyrosine phosphorylation of RPTPe serve to regulate its activity, whereas PTP1B is associated with the endoplasmic reticulum and can be regulated by reversible oxidation and a number of post-translational modifications (Yip et al., 2010). Therefore, RPTPe and PTP1B may regulate LepRb-JAK2 signaling under distinct cellular contexts. Temporal regulation based upon energy status and/or obesity pathology may also regulate PTP specificity as demonstrated by PTP1B and TCPTP’s differential induction by HFD and leptin. Are PTPs differentially expressed across populations of hypothalamic neurons? Given the profound phenotypes in the various conditional, neuronal knockout models, many of the PTPs are expressed in the most commonly studied POMC- and LepRb-expressing cells in the hypothalamus suggesting some amount of overlap. However, careful analysis of PTP expression within defined neuronal populations may reveal important differences in PTP distribution. Unfortunately, the current lack of effective antibodies for immunohistochemical studies has made answering the question of PTP CNS distribution challenging.

Because of their central role in regulating metabolism, PTPs are attractive targets for potential pharmacological intervention in the treatment of obesity and type 2 diabetes. However, designing small molecules to activate or inhibit specific PTPs presents its own challenges. While genetic manipulation in mice produces profound metabolic phenotypes, the metabolic effects of functional changes to PTPs in humans are unclear. Furthermore, attaining drug specificity is a major hurdle given PTPs can regulate different pathways dependent upon cell type. For example, many of the PTPs in this review regulate growth factor-activated pathways in which loss of function could have severe, deleterious tumorigenic effects (e.g., PTEN). PTPs also play a role in non-metabolic CNS functions which could yield potential side effects of drug action. As mentioned previously, SHP2 plays a role in CNS development; therefore, drug action may result in unexpected and unwanted functional changes to neural architecture. PTP1B expression has been shown to affect hippocampal dendritic spine morphology. Interestingly, hippocampus and cortex-specific PTP1B-deficient mice show an enhancement in learning and memory tasks (Fuentes et al., 2012), suggesting PTP1B inhibition may prove fruitful outside of enhancing CNS leptin signaling. Future research is essential for clarifying the metabolic and non-metabolic CNS signaling pathways that are regulated by PTPs in order to advance effective therapeutic development.

Although this review has focused the discussion of PTPs within the context of central leptin and insulin signaling, other pathways also contribute to central metabolism and are regulated by PTPs. Obesity has been described as a chronic inflammatory disease, and elevated circulating cytokines coincide with PTP expression (Loh et al., 2011). Interleukin-6 and ciliary neurotrophic factor have been shown to have leptin-like, CNS-specific weight-reducing effects (Gloaguen et al., 1997; Lambert et al., 2001; Wallenius et al., 2002a,b) and signal through the type-1 cytokine receptor subunit gp130 which has been shown to transduce JAK/STAT signaling (Heinrich et al., 1998). In addition to inflammatory cytokines, brain-derived neurotrophic factor has been shown to affect food intake and body weight (Nakagawa et al., 2002; Lebrun et al., 2006; Unger et al., 2007), and the Trk receptor family have been implicated as substrates of PTPs (Espanel et al., 2002). All in all, PTPs clearly play a role in regulating metabolically relevant central signaling pathways, however many details remain to be clarified. Greater insights into how PTPs attain specificity, what other central functions they have, and what other intracellular pathways they regulate will allow us to thoroughly understand their role in central metabolism.

## Conflict of Interest Statement

The authors declare that the research was conducted in the absence of any commercial or financial relationships that could be construed as a potential conflict of interest.
